# Trench Fever and the Heart

**Published:** 1918-08

**Authors:** 


***A Study of 109 Cases of Trench Fever with Special Reference to the Condition of the Heart.*** By Major T. J. CREAM, R. A. M. C. and Captain B. H. BARTON, R. A. M. C. *Journal of the Royal Army Medical Corps*, March, 1918.

These authors state that apparently there are two distinct types of trench fever.

I. The remittent type with fever of the remittent or intermittent character lasting from five to six days to three weeks or more.

II. The relapsing type with fever for twenty-four to seventy-two hours followed by three to six days apyrexia with abatement of symptoms, then a relapse for 24 to 72 hours, then 3-6 days apyrexia and so on. Cases seldom relapsed with any degree of regularity.

*Symptoms.* The onset, as a rule, is sudden with headache, usually frontal, dizziness, and weakness of the legs, so that the man falls down, severe pain in the legs, usually the shins, sometimes in the thighs and very commonly in the back — belly-ache is also a common complaint. The pains are generally worse at night, constipation is almost constantly present, a slight cough is common.

*Physical Signs* : The pulse is full and bounding — rate 90 to 108; in those cases in which the myocardium is affected it may be of low tension and a rate of 144 or more. The temperature is usually high, 103 to 104 F. The respirations are quiet and unduly increased in frequency. Herpes about the lips and face was noted in several cases. Occasionally there is a slight bronchitis. The heart is quick, normal in cases on the first day, but the myocardium is very commonly affected after a week's illness, there being sometimes definite dilatation. In many cases nothing abnormal was noticed till the patient got up, at which time he had tachycardia and became dizzy and faint, such symptoms persisting unless the patient was put back to bed. The spleen is palpable in some cases. There is often marked tremor when the patients first get up,

*Course* : Of the length of the illness, the authors are unable to speak, with certainty, as the patients were kept only 21 days, when they were discharged to duty, usually of a light character. Regimental medical officers say that the pains in the shins are often very persistent, men complaining of them at night for weeks after their return to duty.

*Treatment* : The course of the disease was not cut short by means of drugs. Salicylate of soda, in increasing doses up to 40 grains, was given every four hours.

The main indications for treatment are :

1. To keep patient in bed until temperature has been normal for 48 hours, continuing to keep a careful watch upon the heart after the patient is up.

2. To keep the bowels open — with calomel and magnesia sulphate.

3. To ease pain and promote sleep.

With reference to the heart, the authors found the relapsing type less liable to affection than those of the remitting type — that the earlier a patient is put to bed, the less likely is his heart to be affected. They conclude by stating that possibly many cases of unstable heart of the soldier have their origin in trench fever, perhaps treated by a day or two off duty.

They have published the article in the hope that D. A. H. may become less common.

